# Comparing health risks and musculoskeletal issues between professional and casual mobile esports players: a cross-sectional descriptive study in Jakarta

**DOI:** 10.3389/fspor.2024.1372979

**Published:** 2024-07-03

**Authors:** Antonius Andi Kurniawan, Kianti Raisa Darusman, Theresia Indriani Prima Chesar, Xarisa Azalia, Erica Kholinne

**Affiliations:** ^1^ Eminence Sports Medicine and Human Performance Center, Jakarta, Indonesia; ^2^ Ophthalmology Service, Eka Hospital, South Tangerang, Indonesia; ^3^ School of Medicine and Health Sciences, Atma Jaya Catholic University of Indonesia, Jakarta, Indonesia; ^4^ Faculty of Medicine, Universitas Trisakti, Gatam Institute Eka Hospital, Jakarta, Indonesia

**Keywords:** video games, musculoskeletal diseases, orthopedic disorder, smartphone, mobile applications, injuries, sports

## Abstract

Mobile gaming poses significant health risks, such as musculoskeletal (MSK) and eye problems, as players maintain the same posture for long periods. The objective of the current study is to describe the prevalence and assess the association of musculoskeletal and eye problems among professional gamers (PG) and casual gamers (CG) with their physical activity level and physical fitness. A total of 94 mobile-gaming athletes (43 PG, 51 CG) were prospectively recruited in this study. Descriptive analysis was performed for BMIs, fatigue scores, musculoskeletal problems profile, and eye problems profile. The associations between physical activity level, BMI, grip strength, flexibility, and MSK injury were tested with a Chi-square test. A total of 65.96% of the players experienced MSK complaints, with the shoulder (26.2%), neck (25.4%), and hand (21.3%) being the top three affected regions. This study also found ocular issues among the players, with eye fatigue (*n* = 50) as the most frequent complaint. Differential outcomes were observed between the PG and CG groups regarding physical activity (*p* = 0.001) and flexibility (*p* = 0.02). Conversely, no statistically significant variances were detected between the PG and CG concerning musculoskeletal (MSK) disorder indicators (*p* = 1.000), body mass index (BMI) (*p* = 0.132), body fat (BF) percentage (*p* = 0.317), grip strength (*p* = 0.006 for the right side, *p* = 0.116 for the left side), or waist circumference (*p* = 0.680). Furthermore, a significant association was observed between physical activity and BMI (*p* = 0.029). Study results showed that two-thirds of players experienced MSK injury, with the most common complaints being neck, shoulder, hand, and wrist pain. All participants reported at least one eye problem, with the majority reporting multiple complaints. There were significant differences observed in physical activity and flexibility between the PG and CG groups; however, no correlation was found between musculoskeletal injury incidence and the players’ physical fitness variables. This lack of correlation may be attributed to the relatively short career span of gamers.

## 1 Introduction

Mobile gaming has gained widespread popularity as both a recreational activity and an Olympic event, with a significant global following (1–3). Indonesia is the world's 16th-largest gaming market and the largest in Southeast Asia. The total revenue obtained from the gaming market at the end of 2021 is 1.92 billion USD. The Southeast Asian esports market is growing mostly due to Indonesia, which accounts for 43% of the region's total number of participants. The vast majority of Indonesian gamers do not own a computer or a gaming console. They are predominantly mobile device (Android) users (4). According to Limelight Networks, Indonesians spend approximately 8.54 h per week gaming, which is somewhat more than the global average (8.45 h per week). In this statistic, Indonesia ranks fourth, trailing China, Vietnam, and India. According to data from the first quarter of 2021, Indonesia was placed second in terms of yearly rise in mobile game downloads (App Store and Google Play). The number of downloads grew by 26% to 790 million. 79% of Indonesians identify themselves as gamers. Almost half of users play every day, and one-fifth play 4–6 times each week, spending 30–60 min each time (4).

Similar to traditional athletes, esports players are prone to overuse injuries. On average, an esports player may spend 5.5–10 h practicing daily, particularly in preparation for competitions. Poor posture can lead to musculoskeletal and ocular disorders, which pose significant issues for esports players (2). The health problems encountered by esports players are akin to those experienced by office workers who spend prolonged periods in front of a computer (5).

Esports injuries and health studies have been focused on computer-based esports, however, there has been little investigation on mobile esports games. The use of handled mobile devices has been proven to cause subclinical and clinical musculoskeletal disorders in the head–neck, shoulder–arm, and hand–thumb areas (6). Several studies have also introduced the impact of increased screen time on ocular disorders such as tearing, tired eyes, blurred vision, burning sensation, redness, and double vision while non-ocular symptoms include neck pain, general fatigue, headache, and back pain (7–9). Esports players are prone to eyestrain, followed by neck and back pain. Apart from that, esports players are also susceptible to wrist and hand injuries such as carpal tunnel syndrome (2, 10).

According to a recent study, more than 90% of millennials prefer to play online games on mobile devices rather than computer-based devices (11). Because of changes in screen size, viewing angle, and finger, hand, and arm movements, mobile gamers adopt different playing positions, engage in various settings, and interact with the gaming environment differently than computer-based players. As a result, over extensive periods of high-intensity training, they may show different postural adjustments, as well as muscle fatigue and tension, as well as repeated injury patterns (1).

A study by Zwibel et al., which investigated computer-based esports, mentioned that more than 25% of players practice more than 5 h per day, and more than 50% of players have a playing duration of more than 2 h until the standing break. Players fix their eyes on the monitor continuously which causes eye fatigue. The study reported that within 30 min of playing, the head shifts forward compared to the spine. The longer the duration of playing, the longer the pressure on the cervicothoracic junction and paraspinal muscles and increases the risk of neck and back injuries. The study also showed that playing video games longer than 3 h is associated with shoulder pain and is likely a result of poor posture while gaming (12). Further research conducted by Lam et al. delved into spinal posture, mobility, and stability among professional mobile esports athletes, revealing significantly poorer spinal posture, diminished mobility, and weaker stability compared to non-athletes. These findings suggest that professional mobile esports athletes are at a heightened risk of developing spinal musculoskeletal disorders, including back and neck pain (13). Another study conducted by Lam et al., which investigated the health profile, fatigue, and musculoskeletal disorders in 50 elite mobile gaming athletes, reported that 34% and 58% of athletes, respectively, experienced frequent and occasional eye fatigue (1).

However, limited information is available regarding the physical and psychological health requirements and the management of injuries among esports athletes. This emerging sport necessitates the involvement of healthcare experts who possess an understanding of social and addictive behaviors, shifts in academic or occupational performance, as well as chronic injuries associated with esports participation, such as wrist or hand ailments, eye strain, and postural evaluations.

The objective of the current study was to describe the prevalence and assess the association of musculoskeletal and ocular problems among professional gamers and casual gamers in Jakarta with their physical activity level and physical fitness. The physical fitness parameters studied in this research are commonly used to assess health-related physical fitness in clinical settings such as body mass index (BMI), body fat percentage (BF), and waist circumference, followed by hand grip strength, and flexibility (14). Additionally, participants were queried regarding their smoking habits, as cigarettes represent the most prevalent form of substance abuse in Indonesia (15, 16). We aimed to ascertain the prevalence of smoking among esports players.

## 2 Methods

### 2.1 Participants

This study included 94 mobile-gaming athletes consisting of 43 professional levels (professional gamers, PG) of two top-tier gaming groups and 51 casual players (casual gamers, CG). PG participated in mobile gaming such as Mobile Legend, PUBG Mobile, and Free Fire. The PG criteria that we included are players who join certain professional esports players organizations whose main profession is as esports players, with a minimum 1 year of playing experience. Meanwhile, the CGs we included are esports players who are not members of a particular professional esports organization, with varying lengths of play.

To determine the appropriate sample size for a study, a power of 80%, a 2-sided significance level of 0.05, and a difference of a 30% incidence rate (42% and 12%) were used. The incidence rate of 42% was based on a previous study conducted by Lindberg et al. in 2020 (17). The study required a minimum of 34 patients in each group to meet the sample size requirement.

### 2.2 Experimental protocol

This study was approved by the ethics committee on 1st March 2023 (IRB 061/KER/FK/III/2023). This research was conducted during the off-season, a period when players are not competing. Data collection occurred from March to May 2023 for professional players and in June 2023 for casual players.

### 2.3 Measurement

Table 1 shows the baseline information collected, including body weight, height, blood pressure, and length of play. Variables that we examined include musculoskeletal and eye problems, smoking habits, physical activity, playing duration, sleeping duration, daytime sleepiness, and physical fitness such as body mass index (BMI), body fat (BF) percentage, hand grip strength, waist circumference, and flexibility.

**Table 1:** Demographic information and items in the supervised questionnaire.

Variables	Value
Age, mean ± SD (years)	24.4 ± 5.1
Age distribution (*n*, %)	
<18 y.o	1, 1.1
18–22 y.o	36, 38.3
22–25 y.o	23, 24.5
>25 y.o	34, 36.2
Body height, mean ± SD (m)	166.0 ± 7.3
Body weight, mean ± SD (kg)	64.7 ± 17.0
BMI, mean ± SD (kg/m^2^)	23.1 ± 6.7
Fat ratio, mean ± SD (%)	21.5 ± 11.6
Gaming duration, mean ± SD (hour)	6.0 ± 2.0
Gender (*n*, %)	
Male	79, 84%
Female	15, 16%
Gaming duration (*n*, %)	
<3 h	41, 43.6%
>3 h	53, 56.4%
Sleeping Duration (*n*, %)	
<6 h	15, 16%
6–7 h	56, 59.6%
>8 h	23, 24.4%
Daytime Sleepiness (*n*, %)	
No sleepiness	46, 49%
Mild	32, 34%
Moderate	15, 16%
Severe	1, 1%
Smoking habit (*n*, %)	
No smoking	48, 51%
Smoking	41, 44%
Former smoker	5, 5%
Physical Activity (*n*, %)	
Low	72, 76.6%
High	22, 23.4%

SD, standard deviation; m, meter; kg, kilogram; BMI, body mass index; y.o, years old.

BMI was categorized into five groups according to the WHO Asian-BMI classification: underweight (<18.5 kg/m^2^), normal weight (18.5–22.9 kg/m^2^), overweight (23–24.9 kg/m^2^), obese I (25–29.9 kg/m^2^), and obese II (≥30 kg/m^2^) (18). We used Tanita Segmental Body Composition Monitor (Tanita Inner Scan BC−545N—Japan) for body fat measurement. Participants wore thin or short-sleeved clothing and stood upright while holding the handrails during the measurement process. Body fat percentage was categorized into 5 ranges: obese: >25% (men), >32% (women); overfat: 20%–25% (men), 28%–32% (women); average fitness: 15%–20% (men), 23%–28% (women); athletic fitness: 10%–15% (men), 18%–23% (women); and exceptional fitness/bodybuilder range: 3%–10% (men), 12%–18% (women) (19).

The hand grip strength test was performed by positioning the participants standing with shoulder adducted with neutral rotation, elbow in 180° extension, forearm, and wrist in a neutral position. Then, the manual dynamometer (CAMRY EH101®, China) was placed in the participant's hand. The handle of the dynamometer is adjusted if required—the base should rest on the first metacarpal (heel of palm), while the handle should rest on the middle of the four fingers. When ready the subject squeezes the dynamometer with maximum isometric effort. The duration of the maximum voluntary contraction for the handgrip test execution was 3 s. No other body movement is allowed. Participants performed the test twice, consecutively, with each hand (first the right hand and then the left hand). There was a 30-s pause between each repetition and a 1-min rest before evaluating the other limb. All participants received verbal support during the execution of the test. The best result from several trials for each hand is recorded (20, 21). We use a numerical rating system to classify the hand grip strength, with 5 denoting excellent, 4 good, 3 regular, 2 poor, and 1 very poor hand grip strength as shown in Table 2 (23).

**Table 2:** Classification of maximal isometric handgrip strength (22).

Classification	Right hand		Left hand		Sum	
	Absolute (kgf)	Relative (kgf/kg)	Absolute (kgf)	Relative (kgf/kg)	Absolute (kgf)	Relative (kgf/kg)
Excellent	>66	>0.84	>66	>0.85	>132	>1.68
Good	59–66	0.76–0.84	58–66	0.74–0.85	116–132	1.49–1.68
Regular	44–58	0.56–0.75	42–57	0.55–0.73	86–115	1.11–1.48
Poor	36–43	0.47–0.55	36–41	0.45–0.54	71–85	0.92–1.10
Very poor	<36	<0.47	<36	<0.45	<71	<0.92

We classified waist circumference into 2 categories: healthy and at risk, based on gender. Women are said to be at risk if their waist circumference is >88 cm and for men >102 cm, regardless of their BMI category. Waist circumference was measured using WHO guidelines (the midpoint between the lower border of the rib cage and the iliac crest) (24).

Flexibility was tested using the sit and reach (SR) test which was performed using the procedures outlined in the American College of Sports Medicine (ACSM) manual. A standard SR box was placed on the floor, by placing tape at a right angle to the 38 cm mark. The participant sat on the floor with shoes on and fully extended one leg so that the sole was flat against the end of the box. Both knees should be locked and pressed flat to the floor—the tester may assist by holding them down. The participant then extended her arms forward, placing one hand on top of the other. With palms down, she/he reached forward sling hands along the measuring scale as far as possible without bending the knee of the extended leg. The hands should remain at the same level, not one reaching further forward than the other. The subject reaches out and holds that position for at least one to two seconds while the distance is recorded (25, 26). The SR test results were divided into 5 fitness categories by age and gender as shown in Table 3.

**Table 3:** Fitness categories for Sit-and-reach test (in) by Age and Sex (27).

Age (year)													
Percentile		18–25		26–35		36–45		46–55		56–65		>65	
Gender		M	W	M	W	M	W	M	W	M	W	M	W
90	Well above average	22	24	21	23	21	22	19	21	17	20	17	20
80	Above average	20	22	19	21	19	21	17	20	15	19	15	18
70		19	21	17	20	17	19	15	18	13	17	13	17
60	Average	18	20	17	20	16	18	14	17	13	16	12	17
50		17	19	15	19	15	17	13	16	11	15	10	15
40	Below average	15	18	14	17	13	16	11	14	9	14	9	14
30		14	17	13	16	13	15	10	14	9	13	8	13
20	Well below average	13	16	11	15	11	14	9	12	7	11	7	11
10		11	14	9	13	7	12	6	10	5	9	4	9

M, man; W, woman.

We also asked participants about their physical activity level using the IPAQ (International Physical Activity Questionnaire). We divided physical activity levels into high, moderate, and low. A participant who scores a high level on the IPAQ engages in at least a moderate-intensity activity for one hour or more every day. Those who score a moderate level engage in at least 30 min a day of moderate-intensity activity on most days. Those who did not fit into the high and moderate categories were grouped into low-level physical activity (28). Moderate-intensity activity is a physical activity that allows a person to talk while doing activities but not sing, examples are walking briskly (3 miles per hour or faster, but not race-walking), water aerobics, bicycling slower than 10 miles per hour on primarily flat or level terrain without hills, tennis (doubles), ballroom dancing, and general gardening. Above moderate-intensity activity, there is vigorous-intensity activity. When doing vigorous-intensity activity, a person cannot speak more than a few words without pausing for a breath. Examples of vigorous intensity-activity are race walking, jogging, or running; swimming laps; tennis (singles); aerobic dancing; bicycling 10 miles per hour or faster that may include hills; jumping rope; etc (29).

### 2.4 Questionnaire

Questionnaires were given to the patient to assess the self-reported musculoskeletal and eye problems. The participants were also given a body diagram, which was presented in the questionnaire. They were required to fill out the Nordic musculoskeletal questionnaire and eye problems in Indonesian (30). The eye problems that we asked about in the questionnaire are blurred vision while viewing the computer, blurred vision when looking into the distance after computer work, difficulty or slowness in refocusing eyes from one distance to another, irritated or burning eyes, dry eyes, eyestrain, headache, tired eyes, sensitivity to bright lights, and eye discomfort (31).

### 2.5 Data analysis

A descriptive analysis was carried out to assess the demographic information, the health profile, the injury characteristics, the number of self-reported eye complaints, and the MSK problems of the participants. The relationships between MSK problems with physical fitness and physical activity were analyzed using a Chi-square correlation test. We utilized the chi-square test to evaluate the association between categorical variables within our dataset, specifically comparing two non-paired groups. This choice of test was made due to the non-paired nature of the data being compared. The factors we analyze from physical fitness are body mass index (BMI), body fat percentage (BF), waist circumference, hand grip strength, and flexibility. We further examined the association between musculoskeletal complaints and demographic factors such as age and gender, alongside exploring the interrelation among physical fitness variables and physical activity. The different incidences of those variables among PGs and CGs were evaluated using the same test. If the chi-squared data assumption was violated, the likelihood ratio was used. All analyses maintained a significance level of *p* < 0.05. SPSS 27.0 software (IBM, Armonk, NY, USA) was employed for all statistical analyses.

## 3 Results

**Figure 1:**
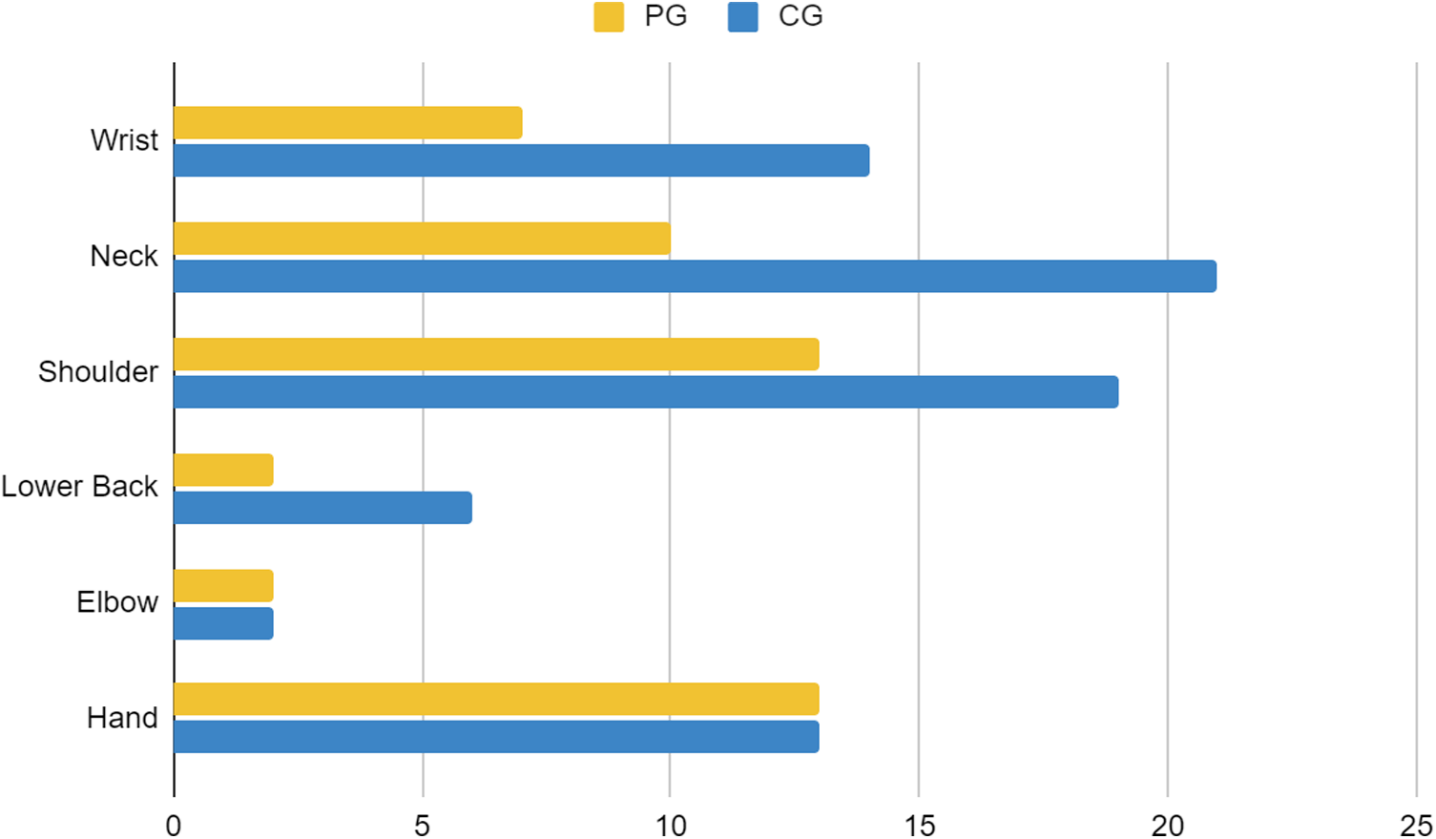
Comparison of musculoskeletal issue body parts between casual gamers (CG) and professional gamers (PG). The X-axis indicates the frequency of reported issues, and the Y-axis represents the different body parts.

**Figure 2:**
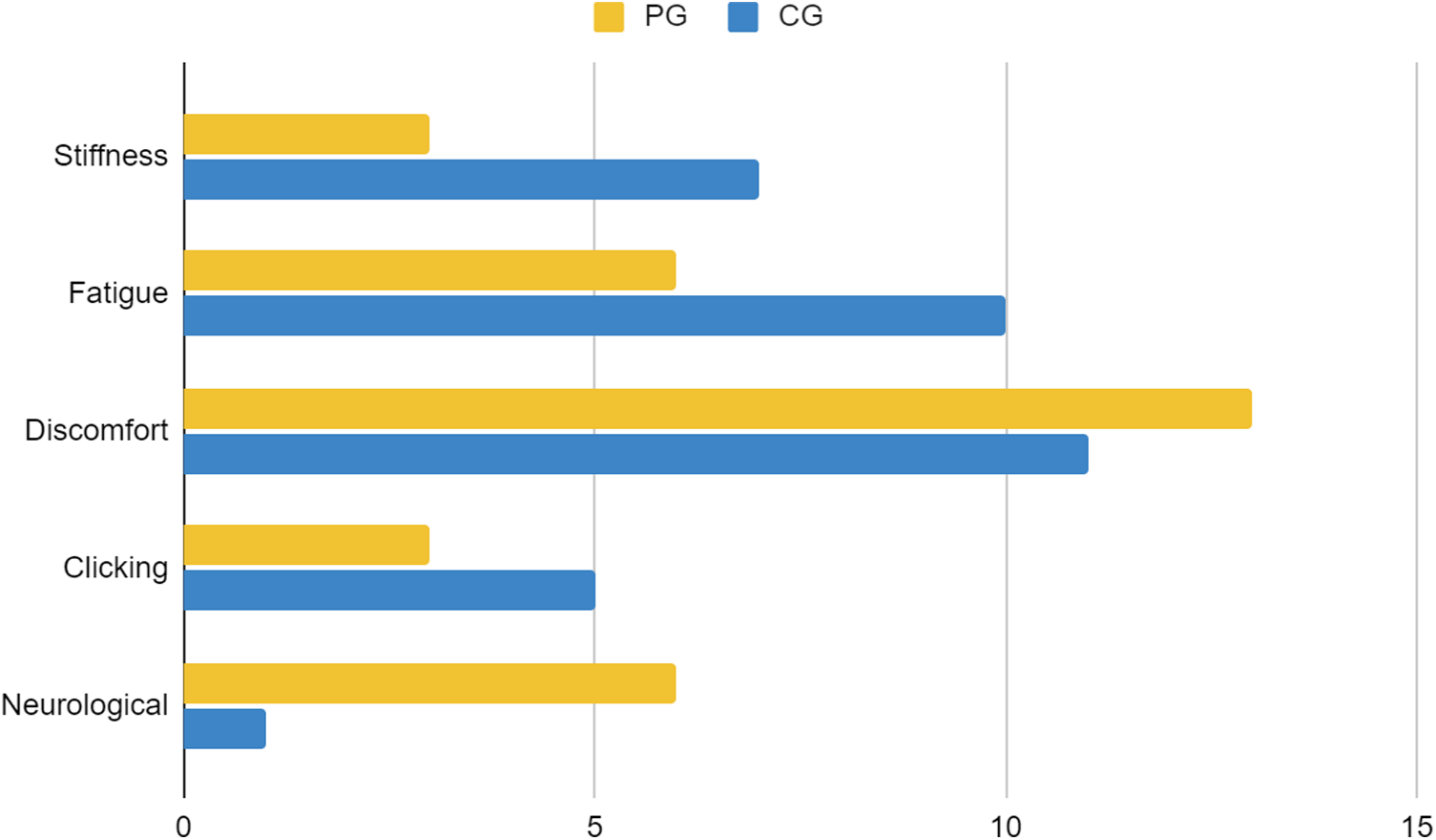
Comparison of types of musculoskeletal issues between casual gamers (CG) and professional gamers (PG). The X-axis indicates the frequency of reported issues, and the Y-axis represents different musculoskeletal issues.

**Figure 3:**
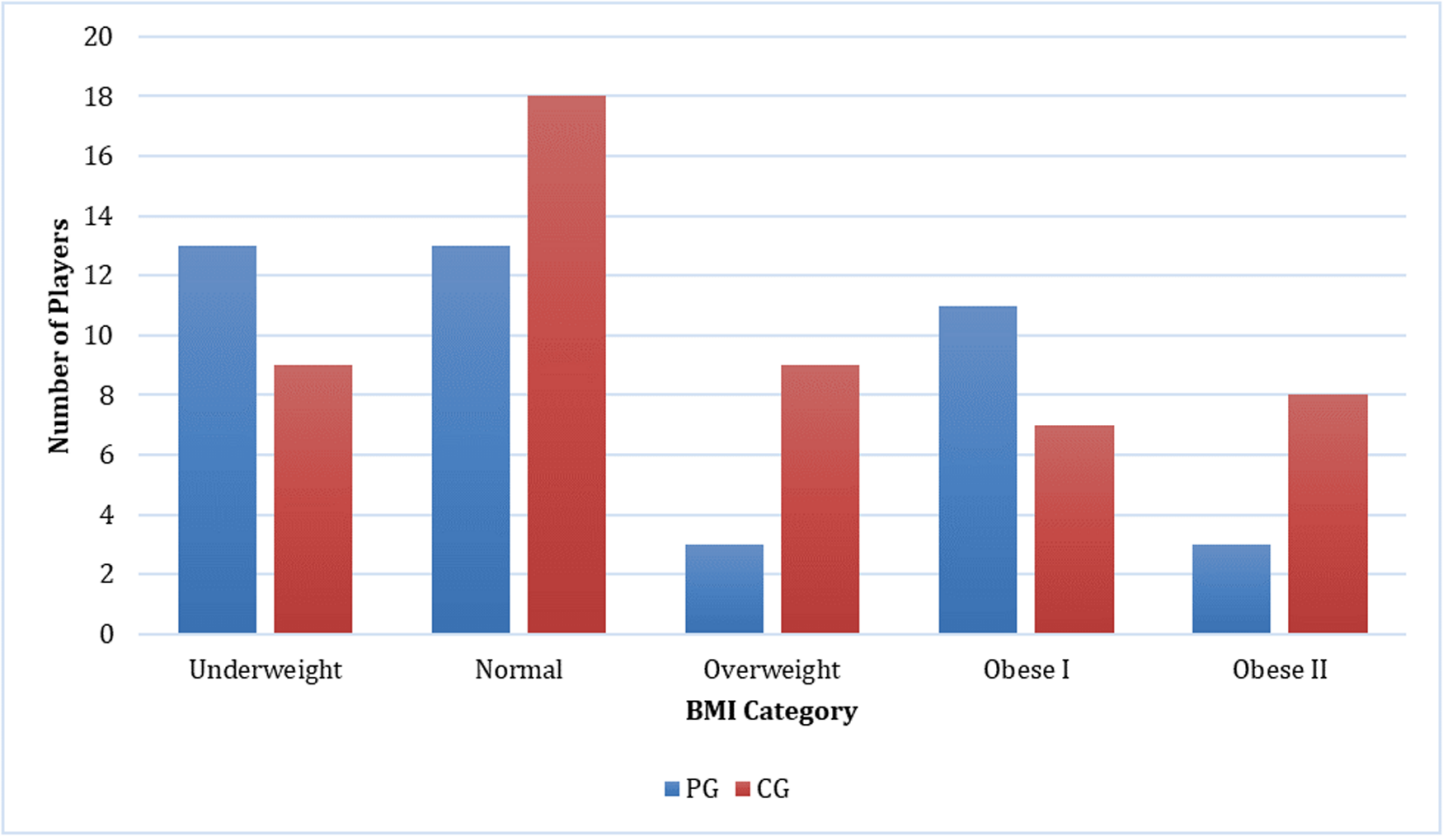
Comparison of body mass Index between casual gamers (CG) and professional gamers (PG). The X-axis represents the body mass index categories, and the Y-axis indicates the number of players in each category.

**Figure 4:**
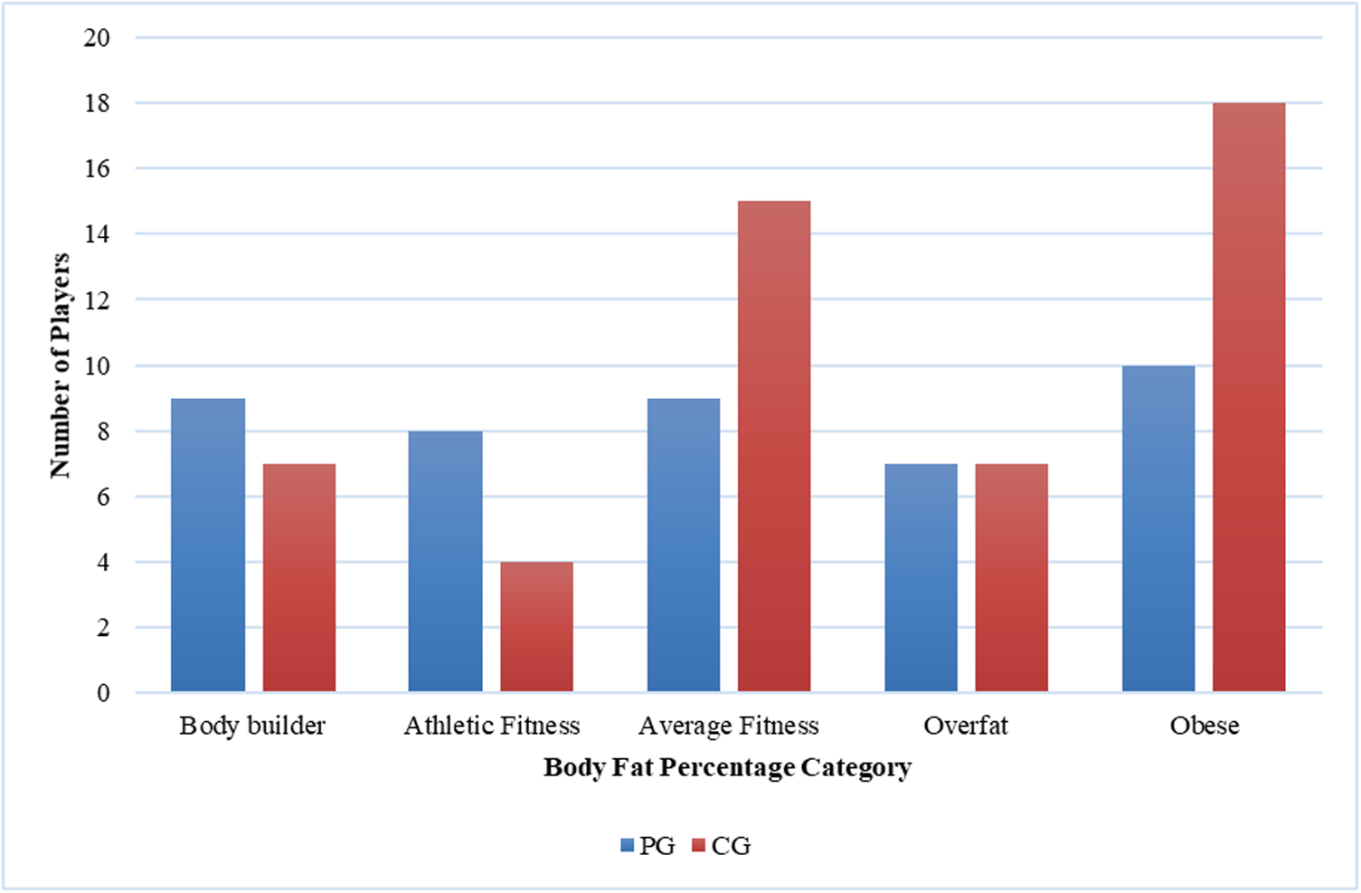
Comparison of body Fat percentage between casual gamers (CG) and professional gamers (PG). The X-axis represents the body fat percentage categories, and the Y-axis indicates the number of players in each category.

**Figure 5:**
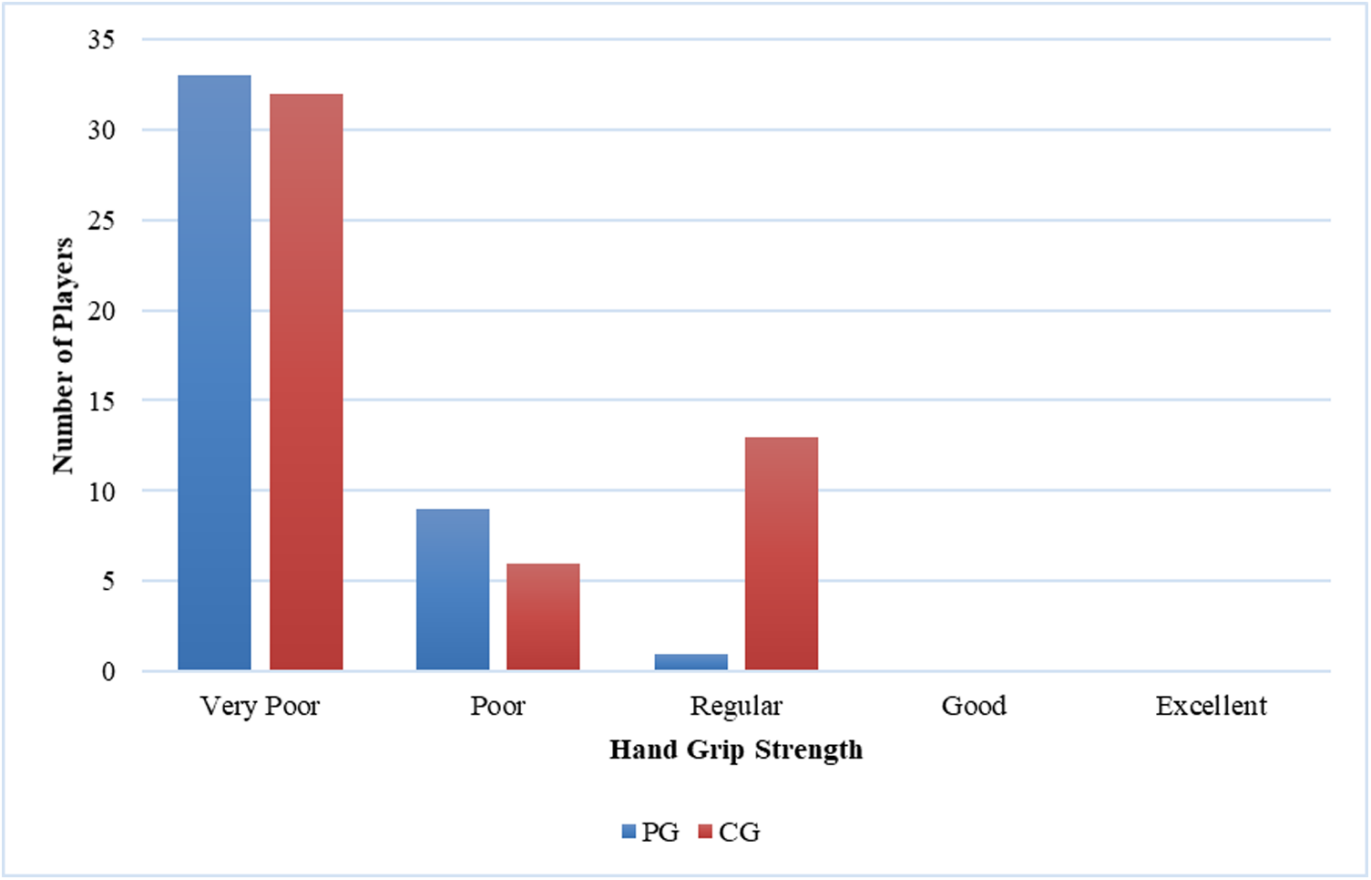
Comparison of right-hand grip strength between casual gamers (CG) and professional gamers (PG). The X-axis represents the hand grip strength categories, and the Y-axis indicates the number of players in each category.

**Figure 6:**
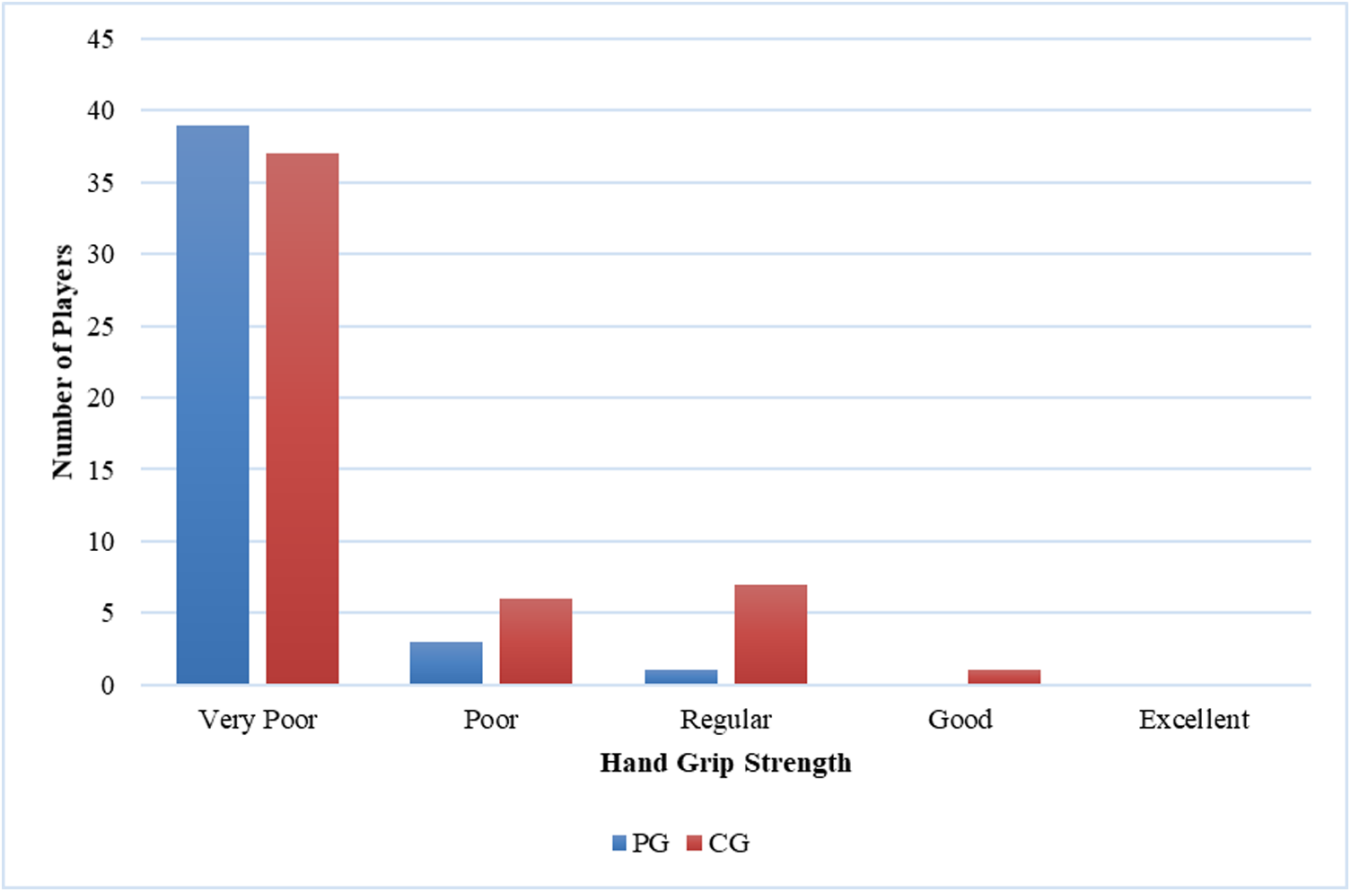
Comparison of left-hand grip strength between casual gamers (CG) and professional gamers (PG). The X-axis represents the hand grip strength categories, and the Y-axis indicates the number of players in each category.

**Figure 7:**
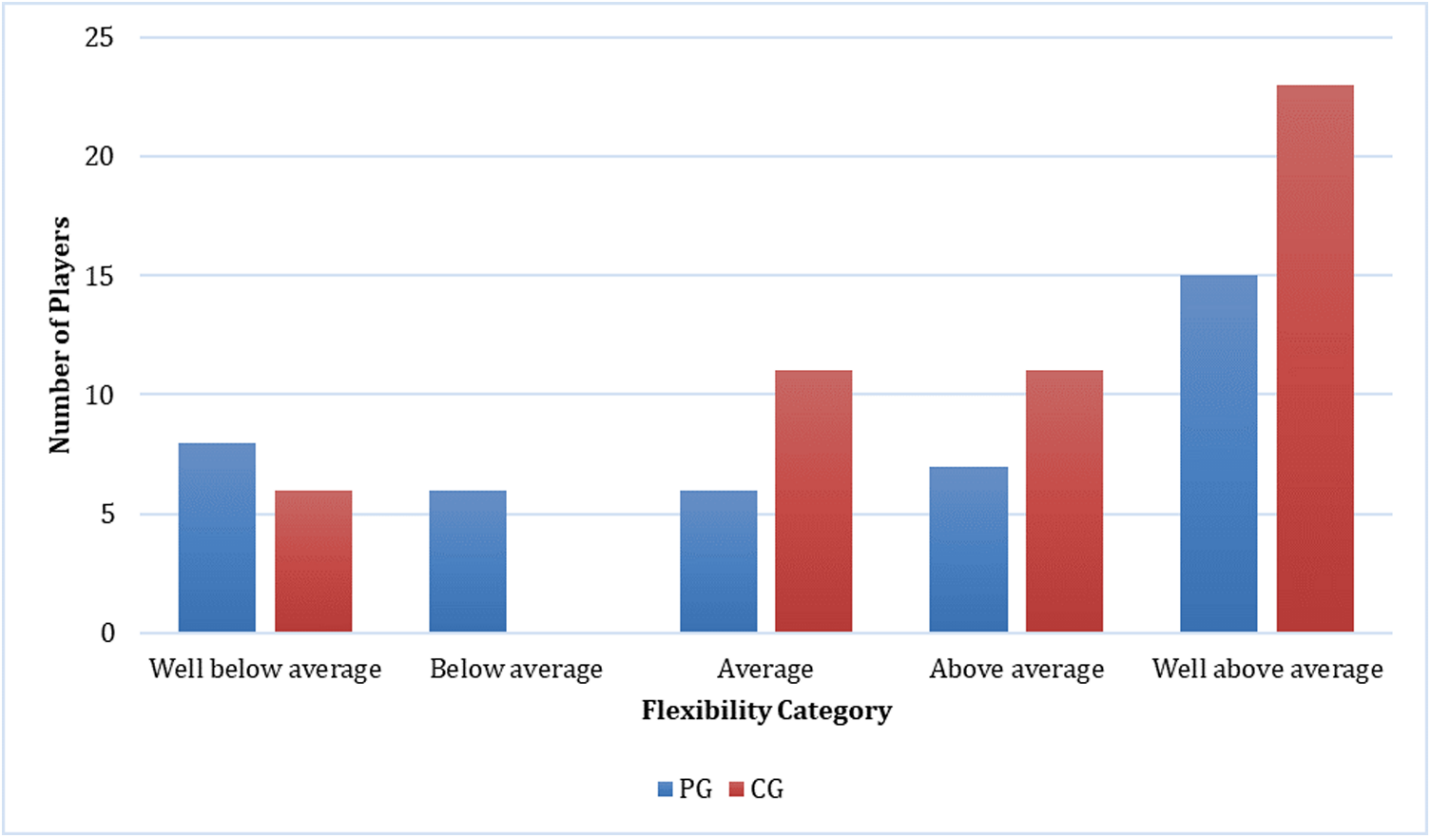
Comparison of flexibility between casual gamers (CG) and professional gamers (PG). The X-axis represents the flexibility categories, and the Y-axis indicates the number of players in each category.

**Figure 8:**
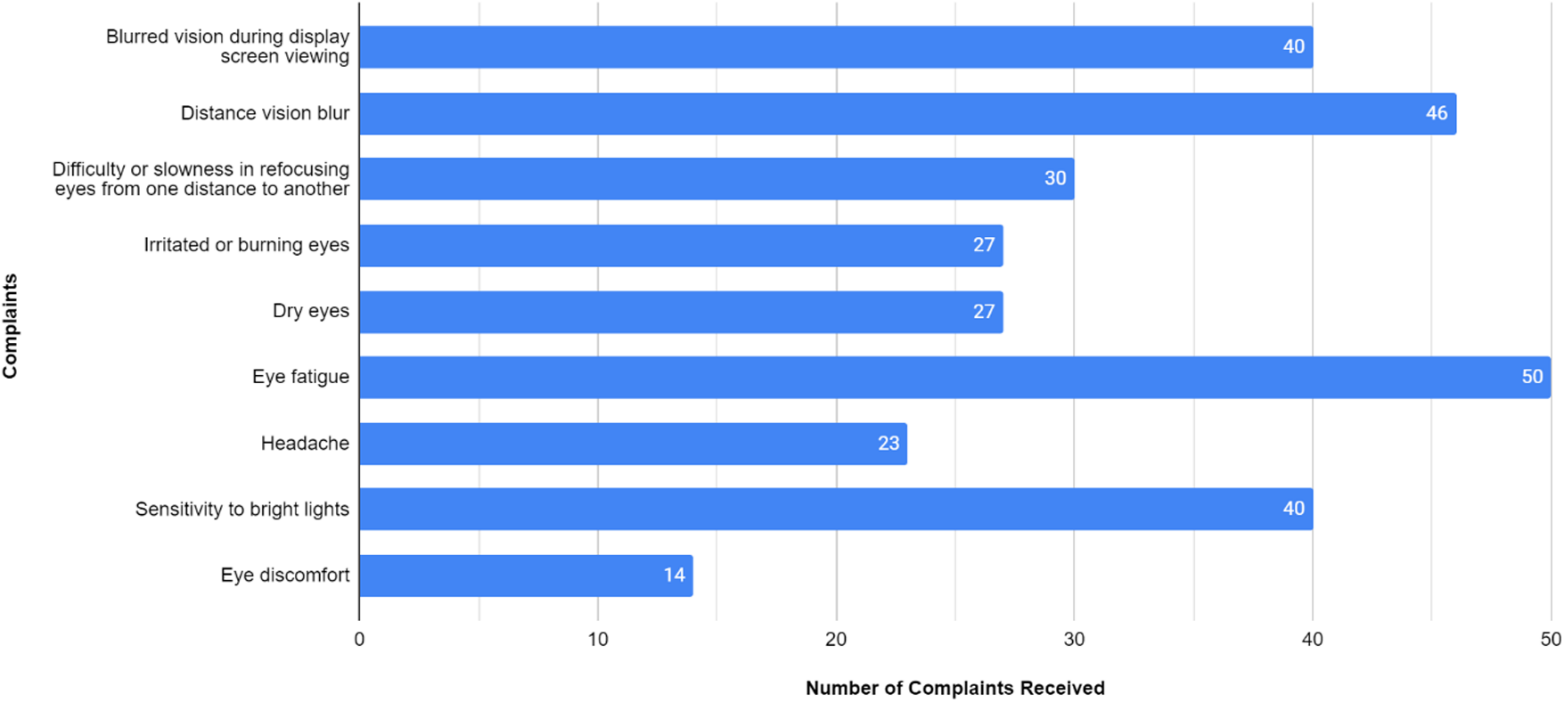
Types of Eye problems Among Mobile gaming athletes. The X-axis indicates the number of players affected by each type of eye problem, and the Y-axis represents the types of eye problems.

For participant demographics, PG consists of 43 players, with 36 players aged under 25 years and 7 players aged 25 years or older. The majority of PG players are male (97.7%, *n* = 42). Within the PG group, 90.7% (*n* = 39) played for more than 3 h, while 9.3% (*n* = 4) played for less than 3 h. Most players slept for more than 6 h (86%, *n* = 37), while the rest slept for less than 6 h (14%, *n* = 6). The majority of PG players reported no daytime sleepiness (51.2%, *n* = 22), while 34.9% (*n* = 15) reported mild daytime sleepiness, and 14% (*n* = 6) reported moderate daytime sleepiness. In the PG group, 62.8% (*n* = 27) were smokers, 34.9% (*n* = 15) did not smoke, and 2.3% (*n* = 1) were former smokers. Most PG participants exhibited low to moderate levels of physical activity (93%, *n* = 40), while 7% (*n* = 3) demonstrated high levels of physical activity. The majority of PG players experienced musculoskeletal complaints (65.1%, *n* = 28). In PGs, injuries were reported in the shoulder (13 cases), hand (12 cases), neck (10 cases), wrist (7 cases), and elbow and lower back (2 cases each) as demonstrated in Figure 1. Discomfort was the most common complaint in PGs with 13 cases reported, followed by fatigue and neurological symptoms with 7 cases each as shown in Figure 2. Regarding eye problems, the most common complaint was blurred vision when looking into the distance after computer work (20 cases), followed by eye fatigue (19 cases), and sensitivity to bright lights (18 cases), details can be seen in Figure 8. In PG, 13 participants had a normal BMI, 13 participants were underweight, 3 participants were overweight, 11 participants were categorized as obese I, and 3 were obese II as shown in Figure 3. Figure 4 illustrates body fat (BF), with most PG players in the obese classification (23.3%, *n* = 10), followed by average fitness as the second largest classification (20.9%, *n* = 9). For waist circumference, 36 PG players had a healthy waist circumference, while 7 were at risk. According to Figure 5, most PGs had very poor right-hand grip strength (76.7%, *n* = 33), while 9 players (20.9%) had a poor rate and 1 player (2.3%) had a regular rate. In Figure 6, most PG players had very poor left-hand grip strength (90.7%, *n* = 39). Three players had poor left-hand grip strength, while 1 player had regular left-hand grip strength. Figure 7 shows that the flexibility capabilities of 15 PG players were in the well-average category, followed by 8 PG players who registered well below average level, 6 below average, 7 average, and 7 above average.

The CG group consisted of 51 players, with 17 players aged under 25 years and 34 players aged 25 years or older. Participants in the CG group consisted of 37 men and 14 women. Within the CG group, 23.5% (*n* = 12) played more than 3 h per day, while 76.5% (*n* = 39) played less than 3 h per day. Regarding sleep duration, 9 (17.6%) CG players slept less than 6 h, while 42 (82.4%) slept for 6 h or more every day. A total of 17 (33.3%) players complained of mild daytime sleepiness, 9 (17.6%) complained of moderate daytime sleepiness, and 1 (2%) person complained of severe daytime sleepiness. Concerning smoking habits, in the CG group, 27.5% (*n* = 14) were smokers, 64.7% (*n* = 33) did not smoke, and 7.8% (*n* = 4) were former smokers. In terms of physical activity, 19 (37.3%) CG players had a high level of physical activity, while 32 (62.7%) had a low to moderate level of physical activity. A total of 34 (66.7%) out of 51 CG players had MSK complaints. The most reported areas of MSK complaints in the CG were the neck (21 cases), followed by the shoulder (20 cases), wrist (15 cases), hand (13 cases), lower back (6 cases), and elbow (2 cases) as presented in Figure 1. Figure 2 shows that discomfort was the most common complaint in the CG, with 12 cases reported. Fatigue and stiffness were ranked second and third, with 10 and 7 cases, respectively. Regarding eye problems, the most common complaint in the CG group was eye fatigue (31 cases), followed by blurred vision when looking into the distance after computer work (26 cases), and blurred vision during display screen viewing (24 cases). The majority of players in the CG had normal BMIs (13 players), 9 players were underweight, and 9 were overweight as shown in Figure 3. Figure 4 demonstrates the BF percentage, most CG players were categorized as obese (18 players), followed by average fitness (15 players). For waist circumference, 42 players in the CG group had measurements within the healthy range, whereas 9 were classified as at risk. Most players in the CG group had very poor right-hand grip strength (62.7%, *n* = 32), 6 (11.8%) players had poor right-hand grip strength, and 13 (25.5%) players had regular right-hand grip strength as presented in Figure 5. For left-hand grip strength, 37 players exhibited very poor strength, 6 had poor strength, 7 had regular strength, and 1 had excellent strength as shown in Figure 6. The flexibility capabilities of 6 CG players were considered well below average, 11 average, 11 above average, and 23 well above average as shown in Figure 7.

The majority of players (66%) reported MSK complaints. The most commonly affected area was the shoulder (26.2%), followed by the neck (25.4%) and hand (21.3%). Among the types of MSK issues, discomfort was the predominant complaint (36.9%), followed by fatigue (24.6%), stiffness (15.4%), clicking (12.3%), and neurological symptoms (10.8%). Eye fatigue emerged as the most common eye problem (*n* = 50) in Figure 8, followed by distance vision blur (*n* = 46), blurry vision during screen viewing, photophobia (both *n* = 40), and accommodative dysfunction (*n* = 30). All players reported at least one eye problem, with many experiencing a combination of issues. Figure 3 revealed that most players had a normal BMI (32.98%), followed by underweight (23.4%) and obese I (19.15%). Body fat (BF), as shown in Figure 4, predominantly classified players as obese (47.72%), followed by average fitness (22.73%). Most participants in both groups had a healthy waist circumference (83%), while the remaining (17%) were at risk. Both PGs and CGs exhibited very poor right-hand grip strength (61.64%) in Figure 5, with variations in poor (20.86%) and regular strength (17.49%). Figure 6 indicated that most players in both groups had very poor left-hand grip strength (80.85%), with some showing poor (9.57%) or regular strength (8.51%). Finally, Figure 7 showcased the flexibility capabilities of PGs and CGs, with variations in categories ranging from well below average to above average.

Differential outcomes were observed between the PG and CG groups regarding physical activity (*p* = 0.001) and flexibility (*p* = 0.02). Conversely, no statistically significant variances were detected between the PG and CG concerning MSK complaints (*p* = 1.000), BMI (*p* = 0.132), BF percentage (*p* = 0.317), grip strength (*p* = 0.006 for the right side, *p* = 0.116 for the left side), or waist circumference (*p* = 0.680). The incidence of MSK injuries was not associated with gender (*p* = 1.000), age (*p* = 0.522), physical activity level (*p* = 0.611), BMI (*p* = 0.223), BF (*p* = 0.139), grip strength (*p* = 0.809 for the right side, *p* = 0.671 for the left side), or flexibility (*p* = 0.229). Furthermore, a significant association was observed between physical activity and BMI (*p* = 0.029).

## 4 Discussion

### 4.1 Musculoskeletal problems

Our data revealed that two-thirds of players experienced MSK complaints, with the most common complaints being pain in the neck, shoulder, hand, and wrist. This correlated with previous research indicating that gamers also experience MSK pain and overuse injuries in the neck, back, shoulder, hand, and wrist (1, 2, 17, 32). Several factors may influence the mechanism of injury in esports players, including poor posture and ergonomics, prolonged static sitting, repetitive upper extremity movements, and lifestyle variables such as physical inactivity (2, 12, 17, 32). Clinically, prolonged use of mobile phones or gaming devices correlates with neck, shoulder, and upper limb pain (1) and also significantly worse spinal posture, mobility, and stability due to prolonged periods of forward flexion head posture (13). Prolonged sitting can trigger abnormal postures that activate the neck and back muscles, leading to muscle strain and fatigue. Weakened muscles are less able to support spinal function, contributing to increased mechanical pressure on intervertebral discs and ligaments, resulting in musculoskeletal pain and discomfort (1). Therefore, in the current study, discomfort was the predominant complaint experienced by most of the players (36.9%) followed by fatigue (24.6%). There is no statistically significant difference in MSK complaints was observed between the PG and CG groups. We also did not observe any correlation between MSK complaints and physical fitness or physical activity among esports players. This finding could potentially be attributed to the relatively brief career span of esports players, typically pursued by individuals in their twenties. Unlike many traditional sports, where athletes may continue competing into their early thirties, esports players often have shorter careers, with approximately one in five professional esports players discontinuing their careers after just two years (33). This trend is driven by the reliance of esports players on their capacity to swiftly and accurately respond to complex visual stimuli, a skill that is believed to decline after the age of 24 (34).

### 4.2 Playing time and physical activity level

The majority of players (76.6%) in the present study report low levels of physical activity. There is a significant disparity in physical activity levels between PG and CG, with PG exhibiting notably lower activity levels compared to their casual counterparts (*p* = 0.001). This is similar to the previous study by DiFrancisco-Donoghue et al. which surveyed 65 collegiate varsity esports players, this study found that the average esports player practices between 5.5 and up to 10 h per day before competitions, 40% of players reported no physical activity outside of gaming, and 15% reported sitting for 3 or more hours without getting up to take a break (2). Another study by Bayrakdar et al. found that average esports players spend 9.3 ± 1.1 h for practice and have poor physical activity levels, recording only 6,646 ± 3,400 steps according to Tudor-Locke & Bassett physical activity level determination criteria (35). Furthermore, it is noteworthy that the guidelines for adult physical activity prescribed by both the American Heart Association and the American College of Sports Medicine are consistent, advocating for a minimum of 150 min of moderate exercise per week, complemented by strength training sessions on 2 or more days per week (36). The majority of our participants failed to meet these recommended activity levels. This aligns with prior research indicating that esports players exhibit significantly lower levels of physical activity than the recommended minimums, with an average frequency of only 1.7 ± 1.9 days per week and a duration of 39.5 ± 40.4 min per day (32, 37).

### 4.3 Sleep duration and sleepiness level

In this study, the majority of players (59.6%) reported sleeping for 6–7 h per night. These findings parallel those of a study on South Korean esports players, which noted delayed sleep patterns, extended wake times, and a nightly sleep duration of less than 7 h (38). The prevalence of late-night gaming among esports players may contribute to their irregular and abnormal sleep schedules. Sleep deprivation can impact esports performance similarly to traditional sports, as sleep plays a critical role in fundamental cognitive functions such as processing speed, attention, and working memory, all of which are vital for esports success. Inadequate sleep not only compromises performance but also undermines physical health and recovery from injuries. Moreover, it heightens the likelihood of resorting to performance-enhancing substances or excessive caffeine consumption, potentially exacerbating the health consequences associated with insufficient sleep (32). Conversely, 49% of participants in our study reported no daytime sleepiness. This disparity may stem from variances in individual tolerance levels to drowsiness, as suggested by experts.

### 4.4 Smoking habits

Our data indicate that 51% of players do not smoke, whereas only 44% of players are smokers, resulting in a slight difference between these two habits. This aligns with a study conducted by Arslan et al., which investigated the esports community at a university in Turkey. The study reported that a notable proportion of esports players, comprising 37.9%, were smokers (39). The rising incidence of smoking among esports players represents a significant concern and exacerbates predisposing factors for stroke (40).

### 4.5 Eye problems

Common eye problems experienced by computer and mobile users can broadly be categorized into two types: (a) dry eye that causes irritation, headaches, and light sensitivity; and (b) visual accommodation problems, such as near-focal blurred vision and difficulty with refocusing (32). In our study, eye fatigue emerged as the most common eye problem (*n* = 50) in Figure 8, followed by distance vision blur (*n* = 46), blurry vision during screen viewing, photophobia (both *n* = 40), and accommodative dysfunction (*n* = 30). This aligns with a previous study by DiFrancisco-Donoghue et al. which concludes that ocular fatigue was the most reported non-musculoskeletal health issue among collegiate esports participants (56%) (2). All esports players reported at least one ocular problem, with the majority reporting multiple complaints. A study by Schary et al. suggests that prolonged visual attention, inconsistent sleep schedules, and excessive computer screen blue light exposure can cause generalized eyestrain and abnormal sleep patterns. It is imperative to implement preventive measures by undergoing regular eye tests, utilizing appropriate glasses, avoiding dry eyes, and taking breaks between games (32).

### 4.6 Physical fitness

#### 4.6.1 Body mass index and body fat percentage

In our study, most of the esports players’ BMI is considered normal. This contrasts with a prior study by Bayrakdar et al., which investigated several athletes from various countries participating in an international competition. The study revealed that the BMI value of esports athletes was 26.03 ± 1.85 kg/m^2^ (35), classifying them as obese according to the BMI assessment criteria. Additionally, Rudolf et al., who examined esports players in Germany, found that the BMI of esports players falls within the fat category, with a value of 24.6 ± 4.8 kg/m^2^ (3). However, in our study, when considering the variable of BF percentage, 30% of the participants were classified as obese. This finding aligns with the research conducted by Difrancisco-Donoghue et al., where only 18% of esports players were classified as obese when assessed by BMI. However, when assessed by BF percentage, it was revealed that 30% of participants fell into the obese category. This suggests that individuals with a sedentary lifestyle may exhibit low muscle mass and a high body fat percentage, which can mask the presence of obesity when BMI alone is considered (37).

#### 4.6.2 Grip strength

In the present study, the test results indicated that a majority of the players performed poorly when tested for right-hand grip strength. Specifically, 69% of the players were classified as “very poor” and another 16% as “poor”. This trend was also observed for the contralateral side, where 81% of players received a “very poor” score and 10% received a “poor” score. This aligns with the low level of physical activity observed in our participants. Prior research suggests that insufficient physical activity may contribute to reduced hand grip strength (41, 42).

#### 4.6.3 Waist circumference

Our data present 83% of gamers had a healthy waist circumference. This may be due to the majority of players having normal BMI. Physical fitness should be evaluated based on each component, including BMI, BF percentage, waist circumference, etc.

#### 4.6.4 Flexibility

For flexibility in this study, 41% of gamers were categorized as well above average, with an additional 19% of players categorized as above average. This may be due to the majority of players were at 18–22 years old which means the flexibility status is still good. Unfortunately, there is no prior research evaluating the flexibility levels of esports players. Our investigation reveals disparities in flexibility between PG and CG (*p* = 0.02). Among professional gamers, 18.6% exhibit well below average flexibility, 14% below average, 14% average, 16.3% above average, and 37.2% well above average. In contrast, within the casual gamer, 11.8% demonstrate well below average flexibility, while 21.6% exhibit average flexibility, 21.6% display above-average flexibility, and 45.1% possess well above-average flexibility. These findings indicate a superior level of flexibility among casual gamers compared to professional gamers. This observation may be elucidated by the comparatively lower level of physical activity among PG in contrast to CG (*p* = 0.001). Our study revealed that a significant portion of professional gamers dedicated at least 3 h daily to esports practice, thereby reducing engagement in other forms of physical activity. Conversely, physical activities such as vigorous exercise are essential for enhancing flexibility, bolstering postural stability, and improving balance, consequently serving as a preventive measure against MSK complaints (43).

Differential outcomes were observed between the PG and CG groups regarding physical activity (*p* = 0.001) and flexibility (*p* = 0.02). There were no significant differences found between the two groups in terms of MSK injury, BMI, body fat, waist circumference, and hand grip strength. The incidence of MSK injuries was not associated with physical activity and physical fitness, also not associated with gender and age. One possible explanation may be the relatively short careers of professional athletes, who typically begin between 16 and 20 years old and may retire around 24 years (34, 44). This was due to the fact esports players heavily rely on their ability to quickly and accurately respond to complex visual stimuli, which may begin to decline after the age of 24 years (34). Another postulation is that, unlike other traditional sports such as basketball, soccer, and badminton, the intensity of movement in esports is lower, resulting in only minor injuries that may not recur easily over the years (1). Additionally, a significant association was observed between physical activity and BMI (*p* = 0.029). Previous studies indicate a strong association between physical activity and BMI (45, 46). Physical activity contributes to increased energy expenditure and plays a crucial role in weight management (45).

### 4.7 Ergonomic and posture improvements

Mobile esports players are susceptible to musculoskeletal (MSK) issues due to ergonomic challenges associated with their playing positions. Typically, players adopt a head-down posture while looking at the screen, hold the mobile device with a bent wrist, and engage in repetitive thumb movements involving twisting or pushing (47). These actions can lead to an imbalance between agonist and antagonist muscles, increasing the risk of muscle injury as discussed in the preceding section (2). Prolonged durations of gameplay and increased flexion of the neck further heighten susceptibility to neck, shoulder, and upper limb pain (48, 49). Additionally, repetitive thumb and finger movements during gaming can result in tendinopathy due to the pushing and twisting motions involved (50).

Musculoskeletal disorders in e-sports players can be reduced by improving posture and work environment. Players need to pay attention to the height and distance of the screen, the grip and hand position, and the use of an arm or backrest (51). The optimal viewing distance for a mobile device is at least 35 cm. However, larger screens, such as those resembling a personal computer (PC), necessitate a proportionally greater viewing distance (52). When using a PC, the center of the monitor should be 5 to 6 inches below the straight vision line at a distance of 20 to 28 inches away. Room light must be adjusted to limit glare. Esports players must be educated to do exercises to prevent eye fatigue such as near-far focusing, palming, and the “20-20-20 rule” that instructs players to look 20 feet away for 20 s every 20 min (12, 53, 54). To reduce neck and back pain, players must regularly do core exercises such as back extensions, rhomboids, balance training, active and passive stretching (54–56). For mobile device usage, to mitigate the risk of neck and shoulder discomfort, it is recommended to engage in stretching exercises targeting the pectoral muscles, upper trapezius, rhomboid muscles, and levator scapulae (57). Adjusting the position of the arms and hands is also important to prevent upper extremity injuries (54).

## 5 Conclusion

We found two-thirds of players experienced MSK injury, with the most common complaints being neck, shoulder, hand, and wrist pain. All esports players reported at least one eye-related complaint, with the majority reporting multiple complaints. Differential outcomes were observed between the PG and CG groups regarding physical activity (*p* = 0.001) and flexibility (*p* = 0.02). There was no correlation between MSK injury incidence and players' physical fitness variables which could be postulated because of the short life span of a gamer. Additionally, a significant association was observed between physical activity and BMI (*p* = 0.029). Future studies should investigate the impact of different levels of mobile game exposure and intensity, and consider implementing preventative measures to promote safe and healthy gaming habits.

## 6 Limitations and future work

This study has several limitations. First, the majority of participants in this study were male, so they are less representative of the general population. Second, we only asked about smoking habits and did not ask about other substance abuse. Future studies are expected to include participants with an equal female-to-male ratio, as well as researching the use of other substance abuse in esports players which can affect players' health and quality of life. In future studies, there is an opportunity to further explore the assessment of eye fatigue through the integration of eye-tracking signals and the detection of muscle fatigue using electromyography among esports players.

## Figures and Tables

**Figure A1:**
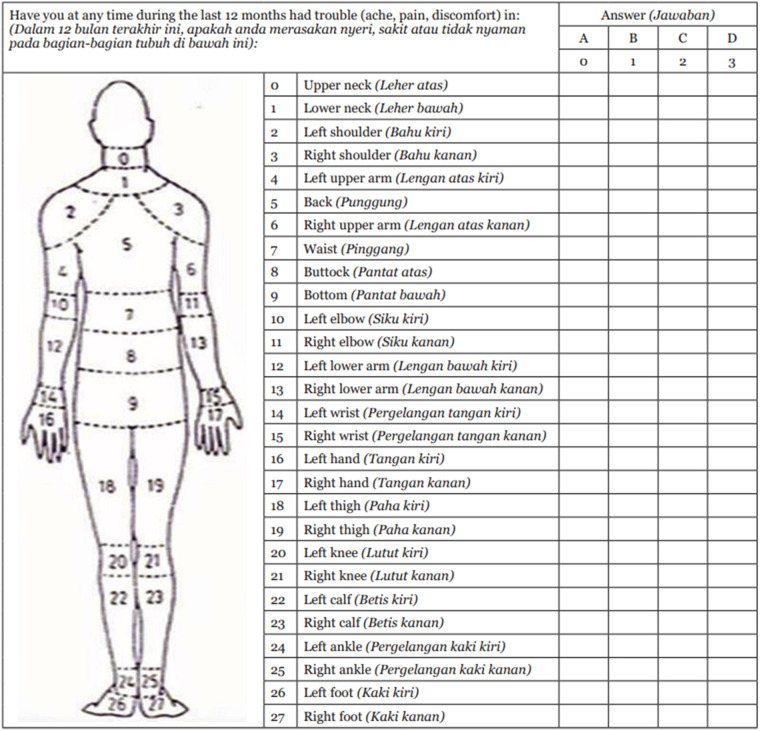
Simplification of the NMQ and translation into Indonesian version (30).

## Data Availability

The raw data supporting the conclusions of this article will be made available by the authors, without undue reservation.
